# Inhibiting Neutrophil Extracellular Traps Protects against Ultraviolet B-Induced Skin Damage: Effects of Hochu-ekki-to and DNase I

**DOI:** 10.3390/ijms25031723

**Published:** 2024-01-31

**Authors:** Issei Inaba, Keiichi Hiramoto, Yurika Yamate, Akihiro Morita, Tomonari Tsutsumi, Hiroyuki Yasuda, Eisuke F. Sato

**Affiliations:** 1 Department of Biochemistry, Faculty of Pharmaceutical Sciences, Suzuka University of Medical Science, 3500-3, Minamitamagaki, Suzuka 513-8670, Japan; sumspp18008@gmail.com (I.I.); hiramoto@suzuka-u.ac.jp (K.H.); yurika727272@yahoo.co.jp (Y.Y.); morita-a@suzuka-u.ac.jp (A.M.); tutumi@suzuka-u.ac.jp (T.T.); 2 Division of Pathological Sciences, Department of Pharmacology and Experimental Therapeutics, Kyoto Pharmaceutical University, Misasagi 5, Yamashina, Kyoto 607-8414, Japan; yasuda20@mb.kyoto-phu.ac.jp

**Keywords:** neutrophil extracellular trap, NETosis, Hochu-ekki-to, histone citrullination, inflammation, UV-B irradiation

## Abstract

UV-B radiation induces sunburn, and neutrophils are pivotal in this inflammation. In this study, we examined the potential involvement of neutrophil extracellular traps (NETs) in ultraviolet B (UVB)-induced skin inflammation, correlating the skin inflammation-mitigating effects of Hochu-ekki-to on UV-B irradiation and NETs. To elucidate NET distribution in the dorsal skin, male ICR mice, exposed to UVB irradiation, were immunohistologically analyzed to detect citrullinated histone H3 (citH3) and peptidylarginine deiminase 4 (PAD4). Reactive oxygen species (ROS) production in the bloodstream was analyzed. To establish the involvement of NET-released DNA in this inflammatory response, mice were UV-B irradiated following the intraperitoneal administration of DNase I. In vitro experiments were performed to scrutinize the impact of Hochu-ekki-to on A23187-induced NETs in neutrophil-like HL-60 cells. UV-B irradiation induced dorsal skin inflammation, coinciding with a significant increase in citH3 and PAD4 expression. Administration of DNase I attenuated UV-B-induced skin inflammation, whereas Hochu-ekki-to administration considerably suppressed the inflammation, correlating with diminished levels of citH3 and PAD4 in the dorsal skin. UV-B irradiation conspicuously augmented ROS and hydrogen peroxide (H_2_O_2_) production in the blood. Hochu-ekki-to significantly inhibited ROS and H_2_O_2_ generation. In vitro experiments demonstrated that Hochu-ekki-to notably inhibited A23187-induced NETs in differentiated neutrophil-like cells. Hence, NETs have been implicated in UV-B-induced skin inflammation, and their inhibition reduces cutaneous inflammation. Additionally, Hochu-ekki-to mitigated skin inflammation by impeding neutrophil infiltration and NETs in the dorsal skin of mice.

## 1. Introduction

The skin is constantly exposed to various environmental stimuli, including ultraviolet rays from sunlight of diverse wavelengths: UVA (320–400 nm), UVB (280–320 nm), and UVC (less than 280 nm). UVB, reaching the Earth’s surface, though potent, facilitates vitamin D synthesis within organisms. It can induce sunburn, DNA damage, skin cancer, compromised immune function, cataracts, photosensitivity, and other maladies [1,2]. UVB radiation causes approximately 80% of the substantial skin damage attributed to sunlight. Concerns have arisen regarding the potential escalation of UVB radiation owing to ozone layer depletion, marked by an increased incidence of skin disorders.

UVB exposure-associated sunburn encompasses erythema and suntan, with erythema being an inflammatory response that manifests several hours post-UVB exposure, causing skin reddening [3,4]. Prior investigations have indicated that the onset of erythema likely involves a defined incubation period post-UV irradiation, suggesting the probable involvement of chemical mediators or cytokines released from other cells, indirectly affecting vascular processes, rather than a direct effect of UVB on blood vessels.

Previously documented evidence underscored the pivotal role of neutrophils in UVB-induced photodamage [5,6,7]. Given the highly vascularized nature of the skin, neutrophils rapidly infiltrate the skin under UVB irradiation. Their recruitment to the sites of skin inflammation exacerbates inflammatory processes and skin injury via the generation of reactive oxygen species (ROS), myeloperoxidase, and elastase. Activated neutrophils have been observed to trigger a distinctive form of cell death called neutrophil extracellular traps (NETs), which release DNA chromatin complexes and potentially worsen inflammation [8,9]. However, the precise role of neutrophil extracellular traps (NETs) in UVB-induced photodamage remains elusive. Strategies aimed at reducing neutrophil counts within skin tissues or suppressing NETs may offer compelling avenues for treating UVB-induced skin damage.

NETs were initially identified as a biological defense mechanism and were acknowledged for their efficacy in combating bacterial and fungal pathogens. Subsequent reports have elucidated the induction of NETs across diverse inflammatory conditions, including thrombosis, autoimmune diseases, and instances of sterile inflammation. The pivotal enzymatic player in the formation of these NETs is peptidylarginine deiminase 4 (PAD4), and the appearance of citrullinated histones, catalyzed by PAD4, is synonymous with the process of NETosis [8,9]. The activation of PAD4 within the cellular nucleus, particularly in response to elevated intracellular calcium, leads to the citrullination of histone H3 (citH3) during NET formation, which in turn induces the decondensation of nuclear DNA, subsequently extruding it beyond the cell-membrane collapse. Consequently, the detection of citH3 is a reliable means of identifying the occurrence of NETs within tissues.

Recently, the attenuation of NET formation has garnered considerable attention as a therapeutic strategy, given the involvement of NETs in various inflammatory conditions. NET inhibition involves the modulation of diverse enzymes and signal transduction pathways within neutrophil cells, and the resultant methodologies for dismantling NETs present promising therapeutic avenues. Physiologically, NETs undergo degradation facilitated by DNase, which is the predominant endonuclease in plasma and is expressed by non-hematopoietic cells. Consequently, numerous researchers have endeavored to leverage DNase I, which exhibits efficient cleavage/hydrolysis of DNA strands in a non-sequence-specific manner. This feature renders it plausible to address NET-dependent inflammatory conditions by administering DNase I to living organisms.

Hochu-ekki-to, a Japanese herbal medicine renowned for its application in allergic diseases, exhibits diverse pharmacological activities, including cytoprotective, antioxidant, immunomodulatory, proangiogenic, and anti-inflammatory effects [10,11,12]. Hochu-ekki-to comprises ten species of medical plants (as follows: Astragali radix (16.7%), *Atractylodes lancea* Rhizome (16.7%), Ginseng radix (16.7%), Angelica Radix (12.5%), Bupleuri radix (8.3%), Zizyphi fructus (8.3%), Aurantii nobilis pericarpium (8.3%), Glycyrrhizae radix (6.3%), Cimicifugae Rhizoma (4.2%), and Zingiberis Rhizoma (2%)) and it was provided by Tsumura Juntendo Co. Ltd. (Tokyo, Japan) and is widely used for various immune-related diseases. Hochu-ekki-to has been reported to suppress the production of reactive oxygen by neutrophils in vitro and its accumulation in inflamed areas [11]. However, the effects of Hochu-ekki-to on neutrophil extracellular traps in inflammatory disease have not been investigated. Moreover, the protective effects of Hochu-ekki-to against UVB-induced skin damage remain unclear. Thus, in this study, we aimed to demonstrate the cytoprotective potential of Hochu-ekki-to against UVB-induced damage in a mouse model and to reveal its potential to suppress NETs.

## 2. Results

### 2.1. Preventive Effect of Hochu-ekki-to and DNase I against UVB-Induced Skin Inflammation 

**Figure 1:**
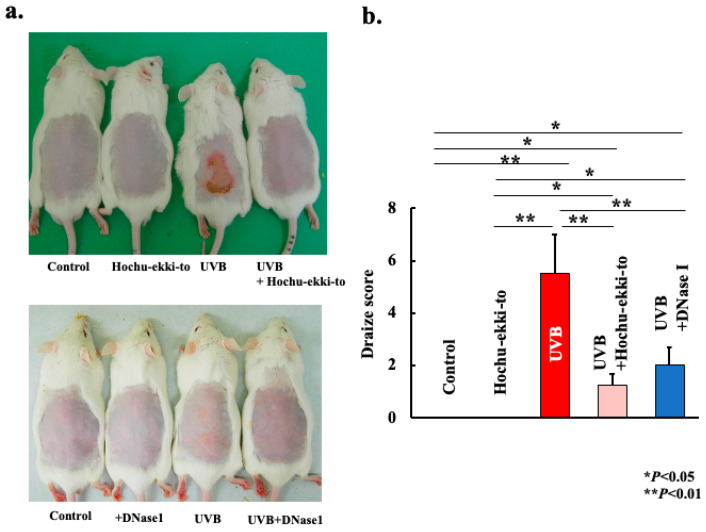
Effect of Hochu-ekki-to and DNase I on dorsal skin dermatitis 5 days after UVB irradiation. (**a**): Representative macroscopic images of the dorsal skin in mice from each group 5 days after the final UVB irradiation session. (**b**): Draize scores of dorsal skin samples from mice 5 days post-final UVB irradiation session. The data are expressed as the mean ± SD of six animals per group. Statistical analysis was performed using ANOVA, followed by Tukey’s post hoc test using the SPSS (version 20) software program (biological replicates; * *p* < 0.05, ** *p* < 0.01).

**Figure 2:**
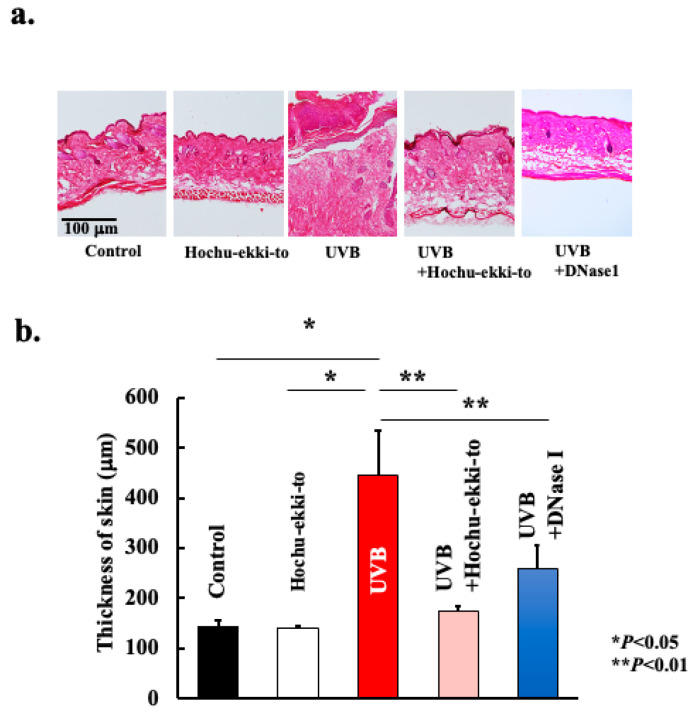
Histological analysis of dorsal skin dermatitis 5 days after UVB irradiation. (**a**): Histological analysis of dorsal skin samples from mice 5 days after UVB irradiation using hematoxylin and eosin staining. The data reflect one typical experiment with six animals per group. Scale bar = 100 μm. (**b**): At the end of the study, the dorsal skin thickness in mice was measured. Statistical analysis was performed using ANOVA, followed by Tukey’s post hoc test using the SPSS (version 20) software program (biological replicates; * *p* < 0.05, ** *p* < 0.01).

Oral administration experiments revealed prominent dermatitis, inflammation, and erythema on the dorsal skin of the mice 5 days after post-UVB irradiation (Figure 1a,c). However, these manifestations of skin inflammation were substantially attenuated in the UVB irradiation + Hochu-ekki-to group (Figure 1a) and + DNase I group (Figure 1c). Furthermore, the Draize assessment, which depicts the extent of erythema and edema on the skin, exhibited a significant decrease solely in the UVB irradiation + Hochu-ekki-to group compared to both the UVB irradiation and UVB + solvent groups (Figure 1b). In the case of DNase I administration, the Draize assessment also exhibited a significant decrease solely in the UVB irradiation + DNase I group compared to that in both UVB irradiation and UVB + solvent groups (Figure 1d). When comparing Hochu-ekki-to group and DNase I group, although there was no statistically significant difference, the Draize assessment score was better with Hochu-ekki-to administration than with DNase I. A histological analysis of dorsal skin samples was performed using hematoxylin and eosin staining (Figure 2a,c). Five days following UVB exposure, the epidermis of the dorsal skin in the UVB irradiation group exhibited increased thickness compared to that in the control group, with notable infiltration of inflammatory cells into the dermis and edema of the dorsal skin. Conversely, these alterations were markedly suppressed in the UVB irradiation + Hochu-ekki-to group and + DNase I group (Figure 2). When comparing Hochu-ekki-to group and DNase I group, although there was no statistically significant difference, the dorsal skin dermatitis was inhibited with Hochu-ekki-to administration than with DNase I. These findings indicated the effectiveness of orally administered Hochu-ekki-to and injected DNase I.

### 2.2. Effect of Hochu-ekki-to and DNase I against Blood ROS Generation after UVB Irradiation

**Figure 3:**
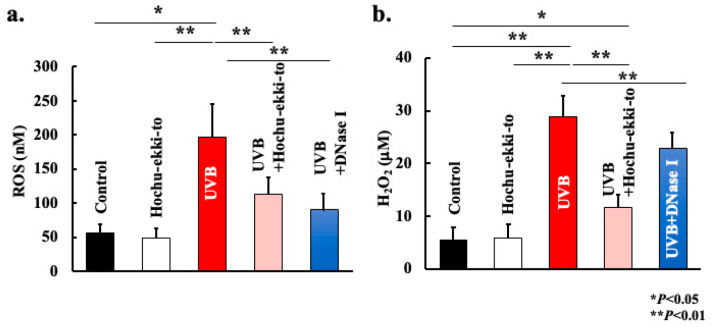
Effect of Hochu-ekki-to and DNase I on reactive oxygen species generation in blood after UVB irradiation. Generation of reactive oxygen species in the blood after UVB irradiation using the OxiSelect In Vitro ROS/RNS Assay Kit. (**a**): Total ROS, (**b**): H_2_O_2_. Statistical analysis was performed using ANOVA, followed by Tukey’s post hoc test using the SPSS (version 20) software program (biological replicates; * *p* < 0.05, ** *p* < 0.01).

We assessed the reactive oxygen scavenging capability of Hochu-ekki-to and DNase I in vivo using the OxiSelect In Vitro ROS/RNS Assay Kit (Green Fluorescence) (Cell Biolabs, Inc., San Diego, CA, USA). As shown in Figure 3, UVB irradiation substantially augmented reactive oxygen production in the bloodstream. Hochu-ekki-to and DNase I demonstrated a profound ability to suppress the formation of ROS and H_2_O_2_ (Figure 3). When comparing Hochu-ekki-to group and DNase I group, their suppressive effect of ROS was the same. However, Hochu-ekki-to group attenuated H_2_O_2_ production more than DNase I group. 

### 2.3. Hochu-ekki-to and DNase I Decrease Neutrophil Migration and Expression of Citrullinated Histone H3 and Peptidylarginine Deiminase 4 in the Dorsal Skin after UVB Irradiation

**Figure 4:**
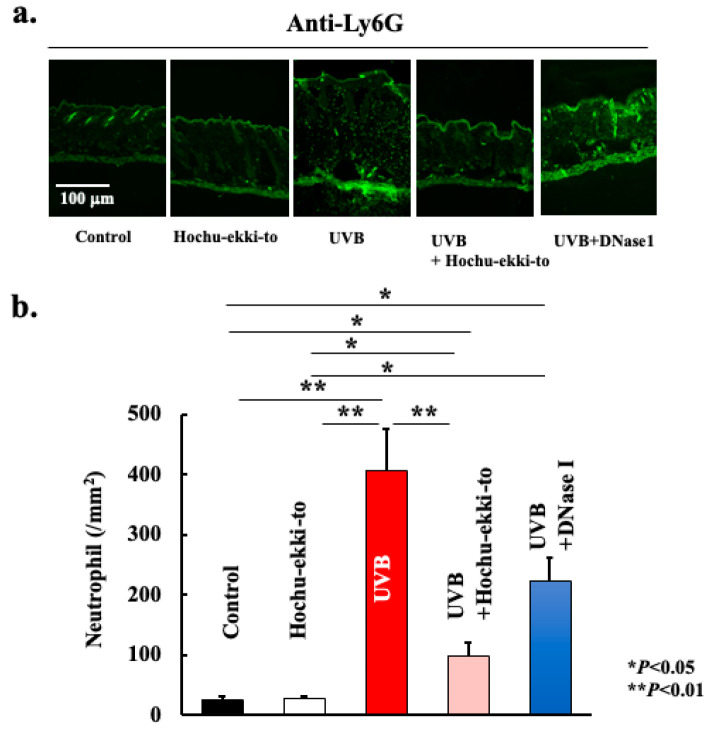
Effect of Hochu-ekki-to and DNase I on the expression of Ly6G (**a**,**b**). Immunostaining was performed to examine Ly6G expression. Statistical analysis was performed using ANOVA, followed by Tukey’s post hoc test using the SPSS (version 20) software program (biological replicates; * *p* < 0.05, ** *p* < 0.01).

**Figure 5:**
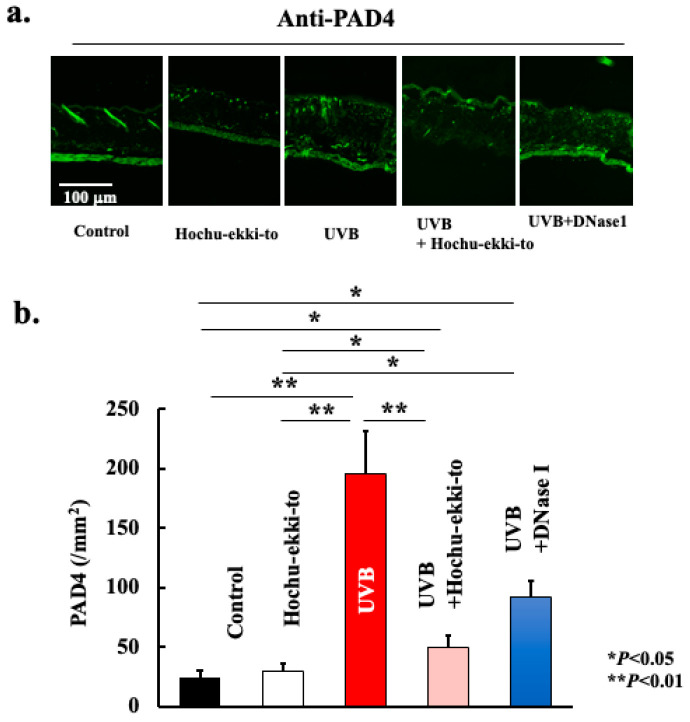
Effect of Hochu-ekki-to and DNase I on the expression of PAD4 (**a**,**b**). Immunostaining was performed to examine the expression of PAD4. Statistical analysis was performed using ANOVA, followed by Tukey’s post hoc test using the SPSS (version 20) software program (biological replicates; * *p* < 0.05, ** *p* < 0.01).

**Figure 6:**
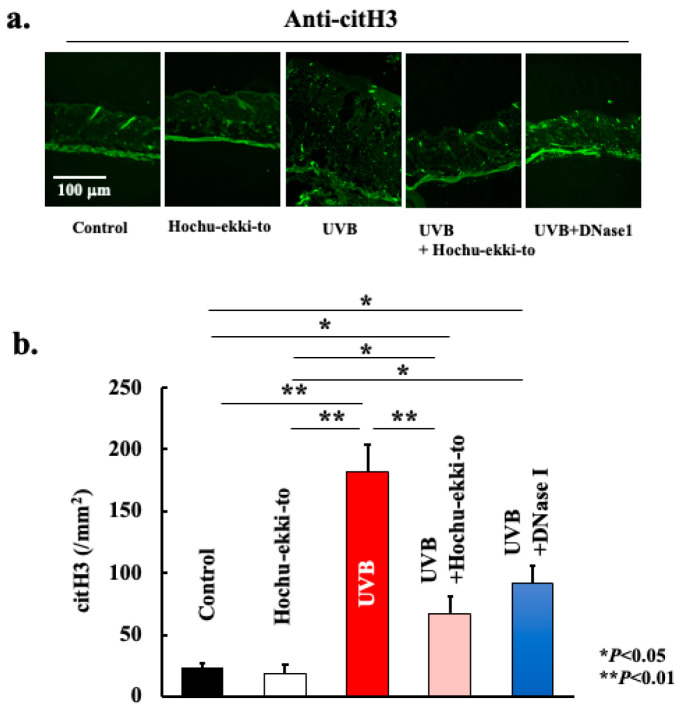
Effect of Hochu-ekki-to and DNase I on the expression of citH3 (**a**,**b**). Immunostaining was performed to examine the citH3 expression. Statistical analysis was performed using ANOVA, followed by Tukey’s post hoc test using the SPSS (version 20) software program (biological replicates; * *p* < 0.05, ** *p* < 0.01).

UVB irradiation-induced neutrophil migration into the skin tissue (Figure 4). Additionally, we analyzed the expression profile of citrullinated histones to ascertain whether the accumulation of neutrophils leads to the formation of NETs. As shown in Figure 5 and Figure 6, neutrophils accumulated in skin tissue after UVB irradiation and expressed PAD4 (Figure 5) and citrullinated histone H3 (Figure 6). Hence, UVB irradiation induced the accumulation of neutrophils in the skin tissue, leading to NET formation. Furthermore, Hochu-ekki-to significantly attenuated neutrophil accumulation and citrullinated histone expression. DNase I also attenuated these events. However, the inhibitory effect of DNase I was lower than that of Hochu-ekki-to. In particular, the inhibitory effect on neutrophil accumulation shown in Figure 4 was lower than that of Hochu-ekki-to. 

### 2.4. Hochu-ekki-to Decreases the Formation of Neutrophil Extracellular Traps of Neutrophil-Like Differentiated HL-60 Cells In Vitro

**Figure 7:**
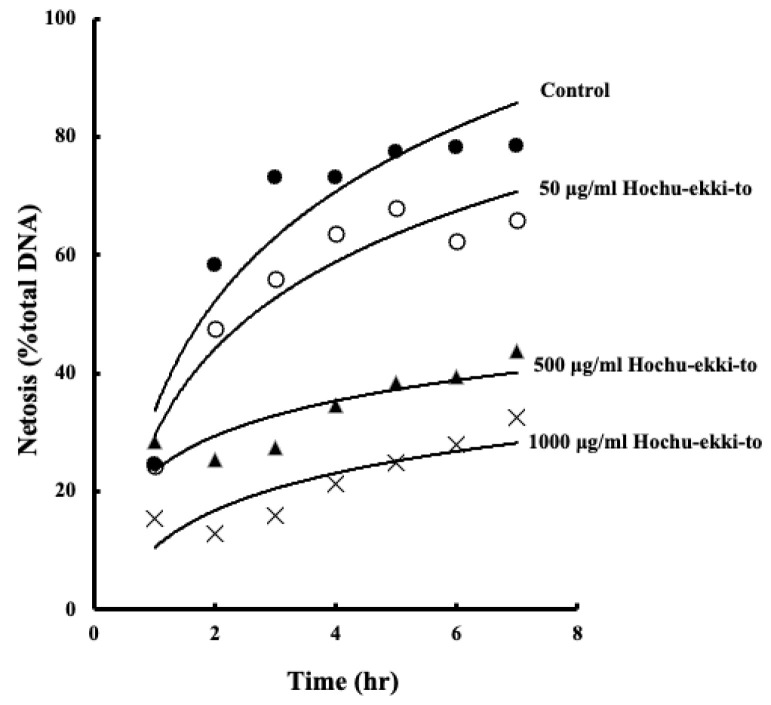
Hochu-ekki-to decreases A23187-induced neutrophil extracellular trap formation (NETosis). After treatment with or without 50, 500, and 1000 ug/mL Hochu-ekki-to for 30 min, NETosis levels in nHL-60 cells induced with 10 μM A23187 for 1 or 6 h were analyzed using a SYTOX green assay. Statistical analysis was performed using ANOVA, followed by Tukey’s post hoc test using the SPSS (version 20) software program (biological replicates).

Hochu-ekki-to inhibits reactive oxygen in the blood and impedes neutrophil migration, suggesting its potential to suppress NETs. Therefore, we employed neutrophil-like differentiated HL-60 (nHL-60) cells, which represent human myeloid leukemia cells, as an in vitro cellular model to ascertain the effects of Hochu-ekki-to on NETs. Consequently, as depicted in Figure 7, Hochu-ekki-to exhibited concentration-dependent inhibition of NETs.

## 3. Discussion

In this study, we demonstrated the involvement of NETs in UVB-induced skin damage. Moreover, Hochu-ekki-to markedly suppressed UVB-induced skin damage by inhibiting neutrophil migration and NETs. Additionally, DNase I administration to disrupt extracellular DNA within NETs significantly mitigated UVB-induced skin damage. Consequently, UVB-induced skin damage may result from extracellular DNA released by NETs.

UVB-induced skin disorders are caused by various factors, among which the pivotal role of reactive oxygen is well documented [13,14,15,16]. As shown in Figure 3, UV-B irradiation substantially augments reactive oxygen production in the bloodstream. It is possible that these reactive oxygen species are generated within the UV-B-irradiated area. During skin inflammation, reactive oxygen may originate from accumulated neutrophils, xanthine oxidase, and mitochondrial sources within UV-B-irradiated tissues [13,14,15,16]. Previous studies have demonstrated that mice lacking NADPH oxidase exhibit partial suppression of UVB-induced skin inflammation [17,18]. In this study, Hochu-ekki-to markedly suppressed both reactive oxygen production in the blood and skin inflammation (Figure 1, Figure 2 and Figure 3). Hence, skin inflammation seems to be related not only to reactive oxygen production from neutrophils, but also to the skin tissue itself, and Hochu-ekki-to mitigates skin inflammation by collectively suppressing these mechanisms. Previous research has shown that Hochu-ekki-to not only suppresses stimulus-induced reactive oxygen production by neutrophils but also inhibits neutrophil migration to inflamed areas [11]. This appears to explain the outcomes of this study.

Neutrophils were absent in the non-inflamed skin layers; however, there was a sudden influx of neutrophils into the skin tissue after stimulation (Figure 4). Skin damage caused by UV exposure or natural sunlight has been reported to signal neutrophil infiltration [18]. This influx of neutrophils into the skin triggers the release of chemotactic factors, leading to the sustained presence of neutrophils within the skin. Previous studies have reported substantial amounts of neutrophil elastase at the sites of UV-induced inflammation [19]. Therefore, considering that neutrophils exposed to UV radiation unfold various mechanisms that potentially damage surrounding tissues and contribute to the effects of photoaging in the skin, it is imperative to acknowledge their ability to induce cell death, which is postulated to generate NETs. In our study, we observed neutrophil infiltration into the skin tissue along with a significant increase in citrullinated histones, a marker indicative of NETs. In vitro, UVB irradiation of human neutrophils induces NETosis [20]. As nearly no accumulation of neutrophils was observed in the normal skin (Figure 4), it is unlikely that early UVB irradiation induces NETosis. It is thought that damage to the skin tissue caused by early UVB irradiation induces the production of skin tissue-derived cytokines, leading to the accumulation of neutrophils. Therefore, NETosis caused by UVB irradiation in vitro appears to occur only partially in the actual tissues. Recently, in vivo studies have reported that NETs may be involved in skin inflammation caused by UVB irradiation [5,21]. These studies used Rho kinase (ROCK) KO mice [21] and Gasdermin E KO mice [5]. Although developing drugs that target these molecules is a promising avenue, the hurdles to their application in treatment are high, and further development is awaited. In this study, we revealed that using the existing Hochu-ekki-to and DNase I, NETs and skin inflammation caused by UVB irradiation can be suppressed, offering hope for its future applications to treat skin inflammation.

Studies have evidenced the direct targeting effect of DNase I on NET degradation [22,23,24,25,26,27]. DNase I effectively degrades the DNA backbone and has been used to treat inflammatory diseases in vivo [22,23,24,25,26,27]. As a constituent of the host defense system, DNase I effectively degrades the DNA backbone within NETs, making it a potential therapeutic target for NETs [22,23,24,25,26,27]. This study illustrates the direct degradation of NET components by DNase I following UV-B irradiation. Our findings are consistent with the therapeutic effects observed in patients with inflammatory diseases treated with DNase I. The results of this study indicate that DNase I can directly degrade NETs upon UV-B irradiation, resulting in a significant reduction in NET levels and tissue damage. Hence, targeting NETs could offer a promising therapeutic approach for treating cancer during UV-B irradiation.

Neutrophil extracellular traps are induced via distinct mechanisms: an NADPH oxidase-dependent extracellular trap, prompted by reactive oxygen production triggered by stimulants such as PMA, and an NADPH oxidase-independent extracellular trap, induced by elevated intracellular calcium levels [28,29]. The in vivo mechanisms governing NET induction are poorly understood, and it remains unclear whether these events occur simultaneously. Previously, skin inflammation caused by UV-B irradiation was shown to be partially suppressed in gp91phox KO mice [17,18]. However, in the current study, this was completely suppressed by the administration of Hochu-ekki-to or DNase I. Hence, extracellular trapping of neutrophils in skin inflammation induced by UV-B irradiation is believed to occur via both NADPH oxidase-dependent and NADPH oxidase-independent pathways. DNase I can eliminate the extracellular DNA induced by these traps, whereas Hochu-ekki-to significantly inhibits both types of neutrophil extracellular traps.

## 4. Materials and Methods

### 4.1. Animals 

All animals were treated in accordance with the animal care regulations of the Suzuka University of Medical Science, and animal experiments were approved by the Suzuka University of Medical Science Animal Experiment Ethics Committee (approval number 31). Specific pathogen-free 8-week-old male ICR mice (Japan SLC Co., Hamamatsu, Japan) were used in the experiments. Under light sevoflurane anesthesia, the dorsal fur of the mice was shaved using electric clippers. 

### 4.2. Induction of UVB Irradiation-Induced Skin Damage

The whole mice body was exposed to UVB irradiation (wavelength range: 280–320 nm, peaking at 305 nm) using a sunlamp (FL-20SE; Toshiba Co., Tokyo, Japan) for 3 days at a dose of 1.0 kJ/m^2^ per day (irradiation time: 45 s/day), with the animals kept under light anesthesia. We filtered out other wavelengths using Kodaul cellulose film (Eastman Kodak Co., Rochester, NY, USA), which is the typical 1 h daytime dose received by humans in Osaka, Japan [30]. To analyze the degree of dermatitis post UVB irradiation, the site was examined for erythema and edema using a modified version of the Draize scoring system, with scores ranging from 0 to 8 [31,32]. The degrees of erythema and edema were determined based on the erythema scores as follows: index value; 0 = no erythema, 1 = very slight erythema (barely perceptible), 2 = well-defined erythema, 3 = moderate-to-severe erythema, and 4 = severe erythema; and edema formation: index value; 0 = no edema, 1 = very slight edema, 2 = slight edema, 3 = moderate edema, and 4 = severe edema (extending beyond the area of exposure).

### 4.3. DNase Ⅰ Treatment

DNase Ⅰ (Roche, Tokyo, Japan) (0.4 mg kg^−1^, intravenous and 2 mg kg^−1^, intraperitoneal) or vehicle (PBS, intraperitoneal) was injected once daily and every other day after UVB irradiation. 

### 4.4. Chemicals 

Hochu-ekki-to was purchased from Tsumura Juntendo (Tokyo, Japan), and its drug composition is summarized in Table 1. The reagent was dissolved in PBS and orally administered once daily (1.0 g/kg/day) and every other day after UVB irradiation. Previous studies [11,33] have reported that 1 g/kg/day is most effective when administered to mice. Therefore, in this study, this dose was used in the administration experiments. The mice were divided into the following four groups: control (non-treated), Hochu-ekki-to, UVB irradiation only, and UVB irradiation + Hochu-ekki-to, with four animals in each group. The vehicle was orally administered once daily and on every other day after UVB irradiation.

### 4.5. Preparation and Staining of Dorsal Skin Sections

On the final day of the experiment, we extracted skin samples under anesthesia. The dorsal skin specimens were fixed in 4% phosphate-buffered paraformaldehyde, embedded in frozen Tissue Tek OCT compound (Sakura Finetek, Tokyo, Japan), and cut into 5 μm-thick sections. These sections were then stained with hematoxylin and eosin in accordance with established procedures for histological analysis of the skin. Skin specimens were stained using antibodies for immunohistological analysis as previously described [34]. The skin specimens were reactive with either mouse monoclonal anti-lymphocyte antigen 6 complex locus G6D (Ly6G: marker of neutrophils) (1:100; BD Biosciences, Franklin Lakes, NJ, USA), rabbit polyclonal anti-citrullinated histone H3 (citH3) (1:100; Abcam, Cambridge, MA, USA), or rabbit polyclonal anti-protein arginine deiminase 4 (PAD4) (1:100; Abcam) primary antibodies. The samples were then washed and incubated with fluorescein isothiocyanate-conjugated anti-mouse and anti-rabbit secondary antibodies (1:30; Dako Cytomation, Glostrup, Denmark). The expression levels of Ly6G, citH3, and PAD4 were immunohistochemically evaluated using fluorescence microscopy. Ly6G, PAD4, and cisH3 were calculated from four random visual fields with constant area using ImageJ software ver. 1.53 (National Institutes of Health, Bethesda, MD, USA). Briefly, the original files were converted to monochrome 8-bit files. Next, the threshold of luminous intensity was voluntarily established.

### 4.6. Quantification of Reactive Oxygen Species via OxiSelect

We utilized the OxiSelect™ In Vitro ROS/RNS Assay Kit (Green Fluorescence) (Cell Biolabs, San Diego, CA, USA) to evaluate the level of oxidative damage in serum 5 days post-UVB irradiation. Samples were loaded into a black 96-well plate, together with varying concentrations of hydrogen peroxide for generating the standard curve, and fluorescence signals were recorded using SpectraMax^®^ (485 nm excitation, 525 nm emission; Molecular Devices Japan, Tokyo, Japan).

### 4.7. Cell Culture

HL-60, a human promyelocytic leukemia cell line (RCB3683; RIKEN BioResource Center, Ibaraki, Japan), was cultured in RPMI 1640 medium (Nacalai, Kyoto, Japan) containing 10% (*v*/*v*) inactivated fetal bovine serum and 1% penicillin/streptomycin (Fujifilm-Wako, Osaka, Japan) [35]. The cells were maintained at 37 °C in a humidified incubator (5% CO_2_), and the culture medium was replaced every 2 days. To differentiate HL-60 cells into neutrophil-like cells, the cells were cultured with 1.25% DMSO for 3 days [36].

### 4.8. SYTOX Green NETosis Assay

NETosis was analyzed using SYTOX green fluorophotometry (Invitrogen, Tokyo, Japan) [37]. The nHL-60 cells were seeded with SYTOX Green, a cell-impermeable nucleic acid dye, in 96-well plates at a density of 5 × 10^4^ cells/well. The plates were divided into four different groups: one group was treated with Hochu-ekki-to, another was treated with 10 μM A23187 (Fujifilm-Wako, Osaka, Japan), the third was treated with 10 μM A23187 and Hochu-ekki-to, and the fourth was the control group (*n* = 6 per group). Concentrations of Hochu-ekki-to were 50, 500, or 1000 μg/mL. After adding 10 μM A23187 to two of the groups, changes in green fluorescence in all the groups were measured every 1 h using a SpectraMax^®^ (485 nm excitation, 525 nm emission; Molecular Devices Japan, Tokyo, Japan). To determine the total DNA concentration, nHL-60 cells were lysed with 1% (*v*/*v*) Triton X-100 (Fujifilm-Wako, Osaka, Japan), and fluorescence changes were recorded. All values were standardized using the total DNA concentration in each experiment.

### 4.9. Statistical Analysis 

All data are presented as the means ± standard deviation (SD) of the values for six animals in each group. Statistical analyses were performed in accordance with the SPSS Institute User Guide. Analyses of variance (ANOVAs) were performed. Significant differences (*p* < 0.05) between means were determined using Tukey’s post hoc test. SPSS software (version 20) was used for the statistical analyses. Statistical significance was set at *p* < 0.05.

## 5. Conclusions

In this study, we observed the involvement of NETs in skin inflammation induced by UV-B irradiation. Moreover, the inhibition of NETs was found to be associated with a reduction in skin inflammation. Furthermore, Hochu-ekki-to was identified to mitigate skin inflammation by impeding neutrophil infiltration into the skin and formation of NETs in the dorsal skin of mice.

## Figures and Tables

**Table 1:** Drug composition of Hochu-ekki-to.

Components	Composition (%)
Astragali Radix	16.7
Astractylodis Rhizoma	16.7
Ginseng Radix	16.7
Angelicae Radix	12.5
Bupleuri Radix	8.3
Zizyphi Fructus	8.3
Auranti Nobilis Pericarpium	8.3
Glycyrrhizae Radix	6.3
Cimcifugae Rhizoma	4.2
Zingiberis Rhizoma	2.0

## Data Availability

The data that support the findings of this study are available on request from the corresponding author.
